# American Medical Association

**Published:** 1884-11

**Authors:** Jno. S. Marshall


### AMERICAN MEDICAL ASSOCIATION.

### SECTION OF ORAL AND DENTAL SURGERY.

### MEETING AT WASHINGTON, MAY, 1884.

REPORTED FOR THE INDEPENDENT PRACTITIONER BY JNO. S. MARSHALL, M. D.,
SECRETARY OF THE SECTION.

(Continued from page 568.)

"Vicarious Catemenial Hemorrhage from the Gums, with Recession of the Gingival Margins and Alveolar Processes, the result of Amenorrhea;" by Dr. W. W. Allport, Chicago, Ill.

SYNOPSIS.

The subject of the report was a young lady aged twenty-five years, of slender form and nervous temperament. As a child she had enjoyed general good health. First menstruated at the age of fourteen and one-half years. For a time the menses were free and unattended with pain, but were from the first delayed from two to four weeks. This condition continued up to the age of twenty, when a period of six months elapsed with no appearance of menstruation. Since that time the menstrual periods have been still more irregular, with gradual decrease in the discharge, and accompanied at times with severe pain. Her health had been considerably broken down, and she was in a nervous, moody condition.

The young lady had been under the professional care of Dr. A. all her life, and he had noticed at about the age of twenty that the margins of the gums were somewhat tumefied and detached from the necks of the teeth, slightly receded, with a tendency to hemorrhage on mastication or when using the tooth-brush. No salivary calculus could be found about the necks of the teeth, or any discharge of pus. Pressure upon the gums over the congested portion, however, caused hemorrhage.

The oral condition at this time was treated by free scarification of the gums and an astringent mouth-wash, and she was advised to consult her family physician with regard to her general health. The case had been seen at frequent intervals during the intervening years, and the same oral treatment temporarily adopted, but with only temporary benefit.

In October, 1883, I was again consulted regarding the condition of her teeth and gums; found them much the same as already described, except the recession had gradually progressed until the necks of the teeth were considerably exposed and the teeth slightly loose in their alveoli. She had grown decidedly anemic in appearance, and complained of her hands and feet being habitually cold. She had pains and weakness in the lower lumbar region, was easily fatigued, and once in every six or seven weeks, corresponding to the commencement of the catemenia, her gums would bleed constantly for from two to three days; the amount was small, but enough to tint the saliva, and this was accompanied with dull headache, increasing languor, and occasionally with epistaxis. The case was referred to the family physician, who upon examination found the vagina quite small, the uterus in a retroverted position with the cervix so antiflexed that he was unable to pass the sound into the cavity of the uterus.

Means were adopted to overcome the abnormal position of the uterus, which have proved entirely successful. During this treatment the Faradic current was used about the pelvis, and the patient given iron and cod-liver oil. After the first week of treatment steady improvement was observed. The first and second menstruations occurring after treatment was begun were delayed till the usual time, but were free and unattended with pain. At the third there was a slight show on the fourth week, but it came freely at the sixth week.

When last seen the patient's appearance gave every indication of being greatly improved. The hemorrhage from the gums has not occurred since the re-establishment of the menstrual flow; the gums have regained their normal color and attachments, except that they had not assumed their normal position at the necks of the teeth.

The mouth is now in a healthy condition, and the result has been accomplished with no other treatment than that adopted by the family physician for the amenorrhea.

DISCUSSION ON DR. ALLPORT'S PAPER.

*Dr. Marshall*—I have recently had under my treatment a case in some respects similar to the one reported by Dr. Allport.

The lady, Mrs. H., thirty-four years of age, was married eight years ago, and two years after gave birth to a son, since which she has been afflicted with a uterine displacement—antiversion—probably the result of subinvolution.

This condition has so affected her general health as to confine her to the house for weeks and months at a time. At every menstrual period she was obliged to keep her bed for from three days to a week, on account of severe suffering. Five years ago she first noticed an inflamed condition of the margins of the gums, and that they were receding from the necks of the teeth. Her dentist was consulted, and he removed a small amount of salivary calculus from about the necks of the teeth, and said that was all he could do for her, as the cause was hereditary.

In June of last year (1883) I was consulted by the lady with regard to the condition of her mouth. She said the recession of the gums had steadily progressed, and on examination I found the gums receded to the extent of about one-eighth of an inch from the necks of the teeth, and quite uniformly about all the teeth in both jaws.

I found but little salivary calculus in any part of the mouth, and what was discovered was upon the roots of the teeth, and completely covered by the gums.

The gums were congested at the margins and loosened from the roots of the teeth to the depth of from a sixteenth to a fourth of an inch.

Pus was found under the gums in those locations where the tartar was present.

The teeth were not loose nor sensitive to percussion, and there was no hemorrhage except when using the tooth-brush.

Upon further inquiry I ascertained the fact of the uterine displacement, and the history of the case as above stated, and that she had lately commenced treatment for that affection. I was of the opinion that there was some association between the uterine trouble and the condition of her mouth, for I could not find a sufficient local explanation to account for the manifestations in the oral cavity. I therefore removed the tartar (sanguinary calculus ?), prescribed an astringent wash for the gums, and told her we would wait the results of the uterine treatment.

I have seen the patient several times since her physician began his treatment, and have noticed a steady improvement in her general health.

She says (March, 1884) the uterine displacement has been entirely overcome, and that menstruation is now normal. The oral conditions are much improved in every respect; the congestion of the gums has entirely disappeared, no pus is to be found about the necks of any of the teeth, and the margins of the gums are firmly attached, but still leaving the necks of the teeth exposed.

This case, like that of Dr. Allport's is interesting, from the fact that the oral affection was but an expression of a disease located in a remote organ.

*Dr. Williams*—I have noticed the same conditions as those mentioned by Dr. Allport and by Dr. Marshall, and cases are not infrequent in which diseased conditions located in the reproductive organs find expression in the oral cavity. One marked case, accompanied by inflammation of the gums and discharge of pus about the necks of the teeth, occurred in a young woman married just a year, and whose mouth was in a normal condition just previous to marriage. It was evidently the result of excessive venery.

*Dr. Stellwagen*—I have seen two cases of vicarious catemenial hemorrhage from the gums and alveolus, occurring after the extraction of a tooth. One was a young lady troubled with a slight ulceration of the *os uteri*. Treatment of the ulceration resulted in the cessation of the hemorrhage.

*Dr. Briggs*—Moved that the discussion on the paper be now passed, and that Dr. Allport be given an opportunity to present the models of a peculiar case of irregularity and the methods adopted for its treatment.

